# Using the General Linear Model to Improve Performance in fNIRS Single Trial Analysis and Classification: A Perspective

**DOI:** 10.3389/fnhum.2020.00030

**Published:** 2020-02-18

**Authors:** Alexander von Lühmann, Antonio Ortega-Martinez, David A. Boas, Meryem Ayşe Yücel

**Affiliations:** ^1^Neurophotonics Center, Biomedical Engineering, Boston University, Boston, MA, United States; ^2^Machine Learning Department, Berlin Institute of Technology, Berlin, Germany

**Keywords:** fNIRS, BCI, GLM, preprocessing, classification, HRF, short-separation, nuisance regression

## Abstract

Within a decade, single trial analysis of functional Near Infrared Spectroscopy (fNIRS) signals has gained significant momentum, and fNIRS joined the set of modalities frequently used for active and passive Brain Computer Interfaces (BCI). A great variety of methods for feature extraction and classification have been explored using state-of-the-art Machine Learning methods. In contrast, signal preprocessing and cleaning pipelines for fNIRS often follow simple recipes and so far rarely incorporate the available state-of-the-art in adjacent fields. In neuroscience, where fMRI and fNIRS are established neuroimaging tools, evoked hemodynamic brain activity is typically estimated across multiple trials using a General Linear Model (GLM). With the help of the GLM, subject, channel, and task specific evoked hemodynamic responses are estimated, and the evoked brain activity is more robustly separated from systemic physiological interference using independent measures of nuisance regressors, such as short-separation fNIRS measurements. When correctly applied in single trial analysis, e.g., in BCI, this approach can significantly enhance contrast to noise ratio of the brain signal, improve feature separability and ultimately lead to better classification accuracy. In this manuscript, we provide a brief introduction into the GLM and show how to incorporate it into a typical BCI preprocessing pipeline and cross-validation. Using a resting state fNIRS data set augmented with synthetic hemodynamic responses that provide ground truth brain activity, we compare the quality of commonly used fNIRS features for BCI that are extracted from (1) conventionally preprocessed signals, and (2) signals preprocessed with the GLM and physiological nuisance regressors. We show that the GLM-based approach can provide better single trial estimates of brain activity as well as a new feature type, i.e., the weight of the individual and channel-specific hemodynamic response function (HRF) regressor. The improved estimates yield features with higher separability, that significantly enhance accuracy in a binary classification task when compared to conventional preprocessing—on average +7.4% across subjects and feature types. We propose to adapt this well-established approach from neuroscience to the domain of single-trial analysis and preprocessing wherever the classification of evoked brain activity is of concern, for instance in BCI.

## 1. Introduction

Brain Computer Interface (BCI) research has gained momentum in the past two decades, fueled by the emergence of increasingly powerful Machine Learning based signal processing methods (Blankertz et al., 2008; Müller et al., 2008; Lemm et al., 2011) and advances in neuroimaging instrumentation. A BCI is an artificial system that bypasses the body's normal efferent pathways, which are the neuromuscular output channels. These systems aim to provide an active interface for communication and control (Birbaumer et al., 1999; Wolpaw et al., 2002) or aim to passively assess covert mental states (Müller et al., 2008; Blankertz et al., 2010) and monitor the “brain at work,” as in the so-called field of Neuroergonomics (Parasuraman, 2003; Parasuraman and Wilson, 2008).

In noninvasive BCI, EEG is currently the primary modality used for both active and passive domains (Birbaumer et al., 1999; Blankertz et al., 2002, 2011; Wolpaw et al., 2002; Dornhege, 2007; Müller et al., 2008; Tomioka and Müller, 2010; Zander and Kothe, 2011; Van Erp et al., 2012; Haufe et al., 2014). However, more recently, an increasing number of studies have explored the suitability of functional Near-Infrared Spectroscopy for BCI and single trial classification of evoked brain activity (Matthews et al., 2008; Hong et al., 2018). fNIRS is a non-invasive, non-hazardous optical imaging technique that measures hemodynamic changes associated with brain metabolism (Villringer and Chance, 1997; Ferrari and Quaresima, 2012; Boas et al., 2014). It uses near-infrared light to measure concentration changes in oxygenated and deoxygenated hemoglobin (HbO and HbR, respectively) in the cerebral cortex and its signals are spatially and temporally comparable to blood oxygenation level dependent (BOLD) signals measured by functional Magnetic Resonance Imaging (fMRI) (Kleinschmidt et al., 1996; Huppert et al., 2005, 2006). The technique has found widespread use both in the research and clinical field despite its low penetration depth and spatial resolution, as it provides good portability, safety, and ecological validity at low-cost and is therefore well-suited for both experimental and real-life settings (Boas et al., 2014; Yücel et al., 2017). Similar to EEG, recent advances in fNIRS instrumentation have led to an increasing number of wearable, light weight, and fiberless systems (Scholkmann et al., 2014; von Lühmann et al., 2015; Zhao and Cooper, 2017) and wearable hybrid EEG-fNIRS systems (Safaie et al., 2013; von Lühmann et al., 2017; Kassab et al., 2018) that help translate BCI research from laboratory environments into real world applications.

**Figure 1:**
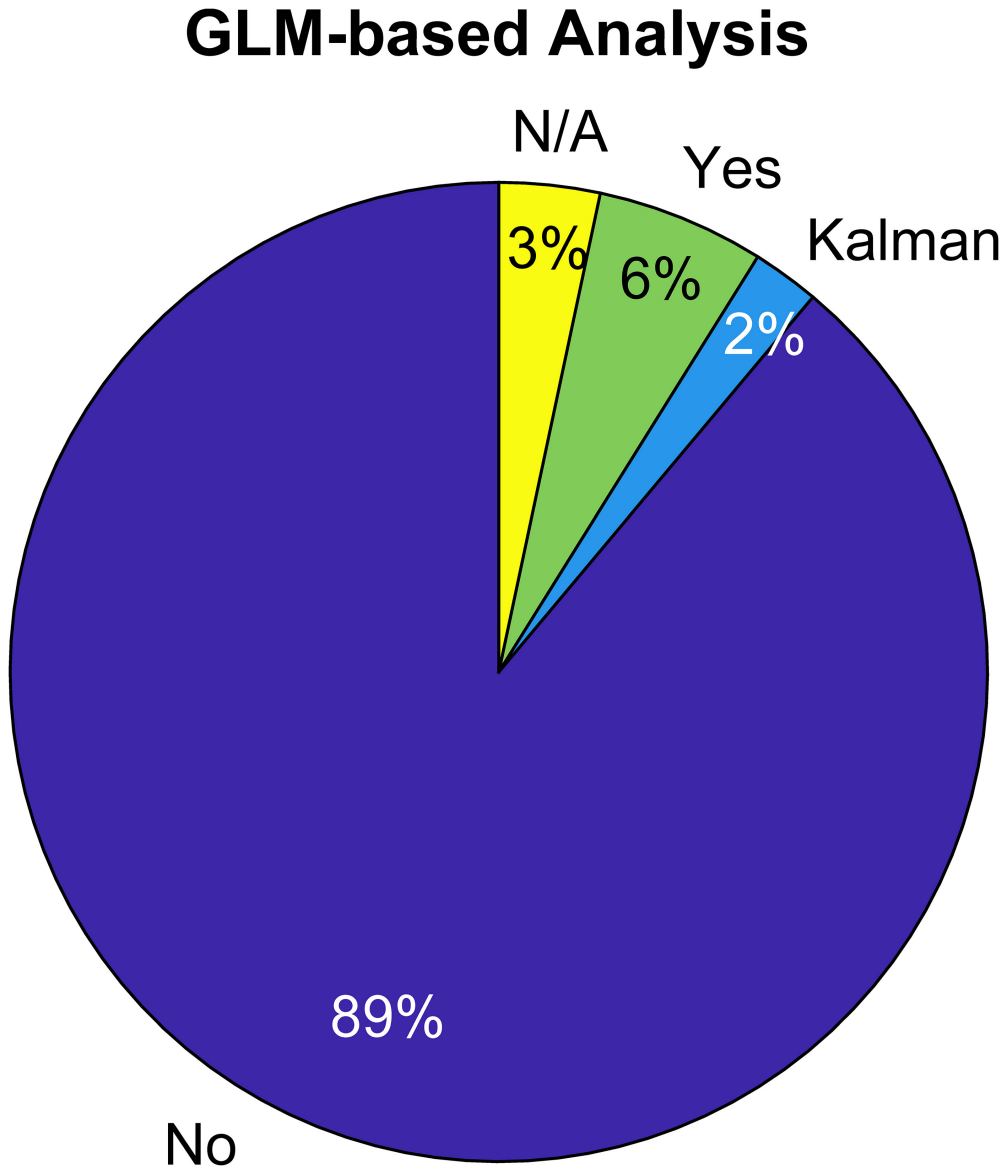
Use of GLM in single trial classification of fNIRS signals (top 100 most cited papers in Web of Science excluding review papers. Keyword search: fNIRS & BCI || fNIRS & Classification).

Due to its dominance in the field, best practice preprocessing recipes exist for EEG to optimize BCI performance (Parra et al., 2005; Blankertz et al., 2008, 2011; Müller et al., 2008), but so far not for fNIRS. Along with the growing number of publications that have studied fNIRS-based BCI and single trial analysis over the course of the last several years (see for instance Naseer and Hong, 2015 for a review), a plethora of methods for optimal feature extraction and classification have been investigated (Matthews et al., 2008; Hong and Khan, 2017; Hong et al., 2018). Remarkably, however, well-established methodology from conventional fMRI and fNIRS neuroscience has so far rarely been adopted for fNIRS single trial signal preprocessing i.e., for removing systemic and non-systemic confounding factors from the signal (see Figure 1). One of these preferred approaches is to use a General Linear Model (GLM) (Friston et al., 1994; Cohen-Adad et al., 2007) which allows simultaneous extraction of the evoked Hemodynamic Response Functions (HRF) while filtering confounding signals with the help of nuisance regressors, for instance short-separation fNIRS measurements (Zhang et al., 2007; Saager and Berger, 2008; Gagnon et al., 2011). By this means, the contrast to noise ratio (CNR) of the evoked hemodynamic brain activity is increased, or in other words the ratio of the brain activity signal to any other physiological or non-physiological signal is increased, and the risk of falsely classifying task-evoked systemic physiology instead of brain activity is reduced. Adopting this approach can therefore significantly enhance accuracy, sensitivity, and specificity of fNIRS single trial classification.

In this manuscript, we first provide a brief overview of the most commonly applied preprocessing steps in the fNIRS-based BCI community, based on statistics obtained from a literature search of the top 100 most-cited papers in Web of Science within the field (search words: fNIRS & BCI or fNIRS & Classification). Then, we introduce the current state-of-the-art analysis in fNIRS neuroscience to fNIRS-based BCI researchers—the General Linear Model with Short-Separation regression (GLM with SS). Thirdly, we provide practical instructions on how to incorporate this approach into any preprocessing pipeline before feature extraction and how to use it within cross validation schemes. This is especially crucial, since learning statistics from the whole dataset by applying the GLM as a “preprocessing step” outside of cross-validation is methodologically wrong and will lead to overfitting. Lastly, we perform a quantitative comparison between the quality of commonly used features when these are extracted from simulated ground truth fNIRS data that was preprocessed either (1) with a pipeline typical for current BCI studies or (2) with the GLM with SS. We show that the GLM-based approach provides better single trial estimates of brain activity, offers a new, more comprehensive feature type, and can significantly improve the classification accuracy in binary classification tasks.

## 2. Preprocessing in fNIRS-Based Bci: an Overview and Perspective

While there have been major advances in fNIRS signal analysis methods since its first establishment, many of them have not yet found widespread use in the wider fNIRS community (Pfeifer et al., 2018). Specifically in single trial analysis and BCI, any fNIRS signal not properly corrected for confounding factors such as systemic interference or motion artifacts can be misleading. A common problem is that machine learning based classifiers exploit any type of task-related information in the signals, including movement artifacts and systemic physiological changes of non-neuronal origin. This can lead to improved discriminability within the designed study but also to a greatly reduced performance when applied outside of the exact same experiment, and is a known pitfall in EEG-based BCI (Müller et al., 2008; Blankertz et al., 2016).

**Figure 2:**
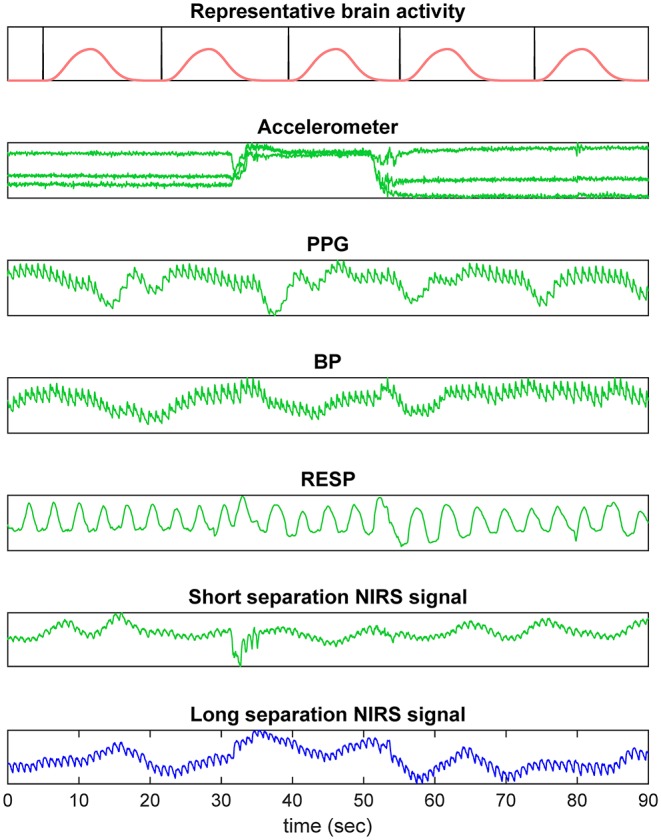
fNIRS signal components. The fNIRS signal is generally a composition of motion related changes, cardiac pulse, blood pressure related changes, respiration, and hemodynamic changes in superficial layers.

fNIRS signals contain two types of noise that contaminate the underlying cerebral hemodynamics: physiological noise and non-physiological noise. Physiological noise involves the systemic interference which is driven by changes in blood pressure due to cardiac, respiration, Mayer waves, and low-frequency oscillations (Elwell et al., 1999; Saager and Berger, 2008; Gregg et al., 2010) or indirectly by head/body movements (von Lühmann et al., 2019), while non-physiological noise involves motion artifacts due to optode-scalp decoupling (Cooper et al., 2012; Brigadoi et al., 2014) and instrumental noise (Figure 2). In order to recover underlying brain activation pattern, one needs to carefully remove these confounding factors from the fNIRS signal. Such correction can either be applied prior to HRF estimation or, ideally, simultaneously with the HRF estimation as in the case of the General Linear Model (Friston et al., 1994; Cohen-Adad et al., 2007), which we will thoroughly discuss in this paper.

The majority of fNIRS-based BCI work performed so far relies on the first approach i.e., preprocessing steps such as channel pruning, removal of physiological noise, de-trending and motion artifact removal/correction are applied to the data prior to HRF estimation. The remaining signal is then assumed to represent the estimated hemodynamic brain response and features are extracted/selected from this signal for classification (Matthews et al., 2008; Hong et al., 2018). We summarize below the most commonly used preprocessing steps currently used in the fNIRS-based BCI field.

**Figure 3:**
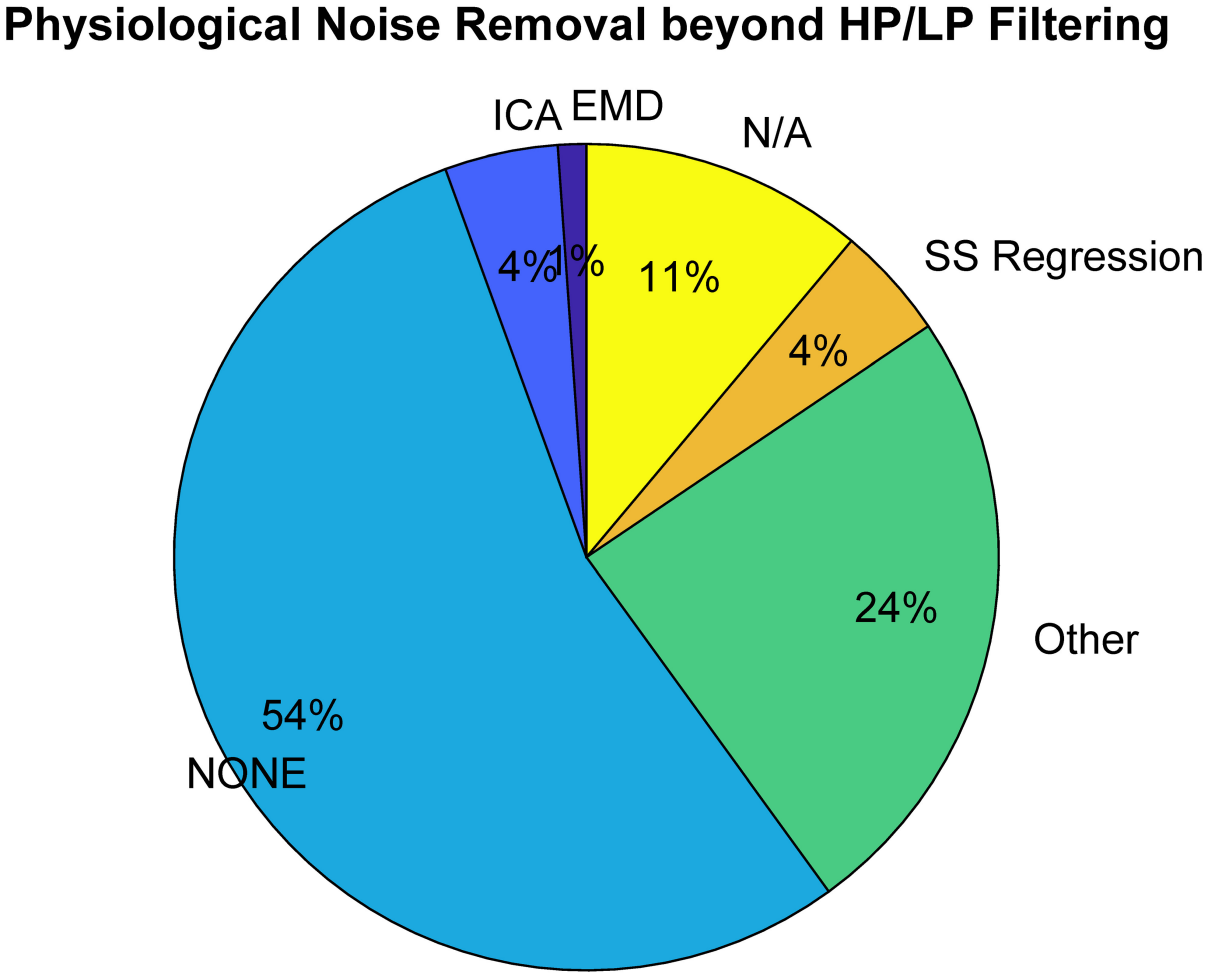
Methods applied for removal of physiological noise beyond conventional bandpass filtering (top 100 most cited papers in Web of Science excluding review papers. Keyword search: fNIRS & BCI || fNIRS & Classification).

1. *Signal quality check and pruning* is the first step in fNIRS preprocessing and is applied to the fNIRS signal regardless of whether the rest of the noise removal is performed prior to or during HRF estimation. In this step, the high frequency components in the signal that are due to non-physiological noise, such as instrumental noise, are typically filtered out and the channels that still have low SNR are removed from further analysis. Among the 100 most cited fNIRS-based BCI studies that we have investigated, ~40% reported applying SNR pruning to their data (see Supplementary Table 1).
2. *Motion artifact correction*. The majority of fNIRS-based BCI studies in our sample do not apply any motion artifact correction to their signal (~80%, see Supplementary Table 1). The remaining studies apply motion correction algorithms typically used in the fNIRS field such as wavelet decomposition (Molavi and Dumont, 2012), spline interpolation (Scholkmann et al., 2010), tPCA (Yücel et al., 2014), CBSI (Cui et al., 2010), or SMAR (Ayaz et al., 2012).
3. *Detrending*. Typically a linear detrending is applied to the relatively long fNIRS signals via high pass filtering or linear least squares fitting across long time windows.
4. *Removal of physiological noise from the signal*. Bandpass filtering is the most commonly used approach in fNIRS-based BCI work to remove the physiological nuisance in the fNIRS signal, particularly very low frequency oscillations and cardiac. Thirty-six percent of the studies reported using only a low-pass filter, 1% reported using only a high-pass filter and the majority reported using band-pass filtering (47%) (see Supplementary Table 1). Certain physiological oscillations such as respiration and Mayer waves fall into the same frequency band as the evoked brain activity in a typical fNIRS experiment, and can therefore not be removed via bandpass filtering without the risk of simultaneously removing signals of interest (Yücel et al., 2016). Thus, additional methods are being employed such as ICA (Independent Component Analysis), EMD (Empirical Mode Decomposition), and CWT (Continuous Wavelet Transform) which decompose the fNIRS signal into (latent) components, with the aim of identifying and removing those components that are due to systemic physiology. In our representative fNIRS-based BCI study sample, the most commonly applied methods for the removal of physiological nuisance signals aside from band-pass filtering (see Figure 3) are ICA (Comon, 1994), EMD (Huang et al., 1998), Transfer Function (TF) models (Pfurtscheller and Florian, 1997), Common Average Reference (CAR) (Pfurtscheller et al., 2010), CWT (Mallat, 1999), and Moving Average Convergence Divergence (MACD) (Appel, 2005). See Matthews et al. (2008), Scholkmann et al. (2014), Hong and Khan (2017), and Hong et al. (2018) for additional methods not mentioned here. ICA is a blind source separation method that assumes statistical independence between non-Gaussian components. The method has the risk of overcorrecting the signal by removing the frequency bands of interest. Results highly depend on the suitability of the applied ICA algorithm for fNIRS signals and methods that exploit sample dependence and higher order statistics are preferable (von Lühmann et al., 2019). EMD is an adaptive method that decomposes the signal into a set of nearly-orthogonal intrinsic mode functions in the time-domain (Huang et al., 1998). The intrinsic mode functions that correspond to the physiological noise in the signal such as cardiac or respiration are then removed from the original signal. Yin and colleagues not only reduce physiological noise using EMD, but also used the intrinsic mode functions as input features for their classifier (Yin et al., 2015). Similarly, CWT decomposes the signal into its components in the time-frequency domain, thus allowing removal of the components that lie in the frequency band of physiological noise. Abibullaev and An removed physiological noise using CWT and used the remaining “de-noised” wavelet coefficients as input features for their classifier (Abibullaev and An, 2012).

The above-mentioned methods can serve their purpose well when there is no additional information on physiological noise available. Ideally, however, independent measures of systemic physiology are acquired along with fNIRS recordings such as respiration or blood pressure variations. The majority of the physiological nuisance signals in fNIRS stems from the superficial layers i.e., scalp and skull (Zhang et al., 2007). Consequently, an independent measure of the hemodynamic changes in superficial layers using a short-separation detector measurement has been proposed (Saager and Berger, 2008). Using Monte Carlo simulations, Zhang and colleagues showed the benefit of using short-separation measurements as reference in an adaptive filter to remove the systemic interference in the long-separation measurements (Zhang et al., 2007). The short-separation measurements and other simultaneous and independent measurements can also be used as regressors to model systemic interference in a General Linear Model framework. This allows simultaneous estimation of brain activity and systemic interference and other nuisance terms in the signal without the risk of removing the underlying brain signal, thus providing a more accurate and robust unbiased estimate of the hemodynamic changes (Diamond et al., 2006; Tachtsidis et al., 2010). While the use of short-separation signals has been shown to significantly improve the robustness of the estimation of hemodynamic response emerging from brain (Gagnon et al., 2011; Yücel et al., 2015), only 4% of the recent fNIRS-based BCI studies used short-separation measurements in their work (see Figure 3) and none applied it in a GLM framework which is the standard approach in neuroscience research today. Some other works, on the other hand, applied the GLM, albeit without short-separation regression (such as Qureshi et al., 2017), and as a preprocessing step on the full dataset.

**Figure 4:**
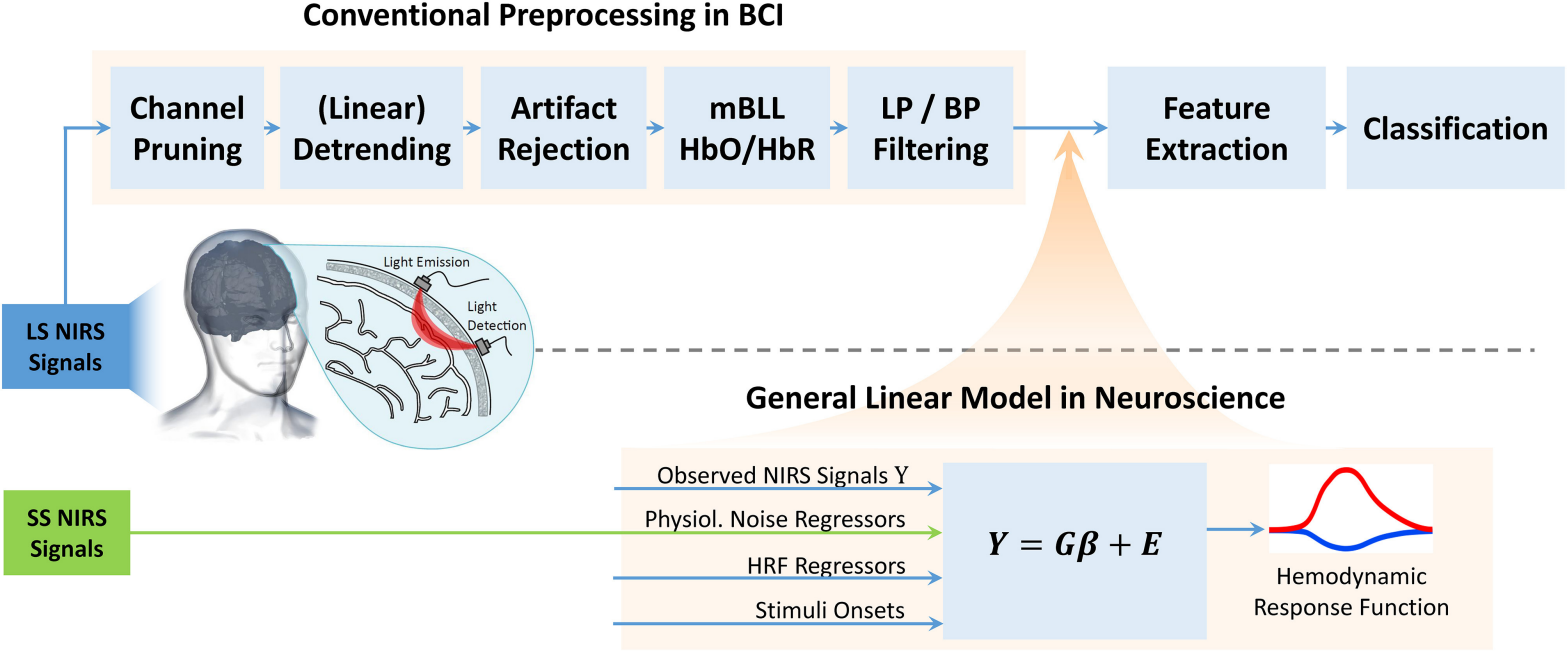
Typical BCI preprocessing pipeline for fNIRS: Linear detrending, pruning of channels with low SNR, artifact rejection, conversion to HbO/HbR with modified Beer-Lambert Law (mBLL), low-pass (LP), or band-pass (BP) filtering. Sequence of blocks can deviate. (Down) established General Linear Model in fNIRS and fMRI Neuroscience using the current best practice: short-separation regression (GLM with SS). We propose to include the GLM into BCI approaches for enhanced performance by using better estimates of the hemodynamic response in fNIRS. Please note that drift removal (detrending) is part of the GLM when polynomial regressors are provided.

In the following section we give a brief introduction to the GLM and show how to correctly integrate it into a conventional BCI preprocessing pipeline (Figure 4) to improve classification performance while strictly avoiding overfitting pitfalls.

*fNIRS BCI studies can benefit from the General Linear Model framework which allows simultaneous estimation of brain activity and concurrent physiological and non-physiological variations, providing a more accurate and robust recovery of the hemodynamic response*.

## 3. GLM Based fNIRS Preprocessing (HRF Regression)

In the following section, we provide a brief introduction to the GLM for fNIRS and show how one can incorporate physiological and non-physiological nuisance regressors into the GLM. This model is discussed in detail in Gagnon et al. (2011) and von Lühmann et al. (2020).

### 3.1. Introduction to the GLM

The General Linear Model represents measured data as a linear mixture of *M* functionally distinct processes (components). We express the GLM for fNIRS as

(1) $$\begin{array}{l} Y=G\;\beta +\text{E} \end{array}$$

where *Y* ∈ ℝ^*T*×*N*^ is the observation matrix with acquired fNIRS data from all time points *T* and recorded channels *N*. We will denote observed data samples of *Y* at time point *t* and channel *n* with scalars *y*_*n*_(*t*), the column vectors of the observation matrix as $y_{n}\in \;{\mathbb{R}}^{T}$ and its row vectors as *y*(*t*) ∈ ℝ^*N*^. *G* ∈ ℝ^*T*×*M*^ is the *design matrix* that incorporates *a priori* knowledge about the expected shape of the evoked hemodynamic response, time structure of the experiment and regressors for drifts and/or physiological nuisance signals. β ∈ ℝ^*M*×*N*^ represents the set of weights for the regressors that model functional brain activity and physiological and non-physiological confounding components. These weights are to be estimated. Components that are not explained by the model are in the additional residual/noise term *E* ∈ ℝ^*T*×*N*^.

Under the GLM assumption, the observed hemodynamic signal *y*_*n*_(*t*) in each of the N channels is modeled by a combination of functional, physiological, and drift components plus the residual:

(2) $$\begin{array}{l} y_{n}\left(t\right)=y_{n}^{functional}\left(t\right)+y_{n}^{physiology}\left(t\right)+drift_{n}\left(t\right)\;+\varepsilon_{n}\left(t\right). \end{array}$$

Both in BCI classification scenarios and conventional neuroscience, it is typically assumed that the evoked hemodynamic signal $y_{n}^{functional}$ is stationary across trials (stimuli δ_*k*_) of the same condition within an experiment and subject. In the GLM, it is modeled either as a canonical HRF using a gamma-variant function (Abdelnour and Huppert, 2009) or with a weighted set of temporal basis functions *b*_*i*_(*t*) made from a linear combination of *H* normalized Gaussian functions *b*_*i*_ = (Δ*t*·*h*, σ), with a standard deviation σ and means separated by Δ*t*, both typically in the order of 0.5 s:

(3) $$\begin{array}{l} hrf\left(t\right)=\sum_{h=1}^{H}b_{i}{\left(t-\Delta t\cdot h\right)\beta }_{h} \end{array}$$

and *hrf* (*t*) is repeated at each stimulus onset δ_*k*_

(4) $$\begin{array}{l} y_{n}^{functional}\left(t\right)=\sum_{k=0}^{K}hrf\left(t\;-\delta_{k}\right). \end{array}$$

**Figure 5:**
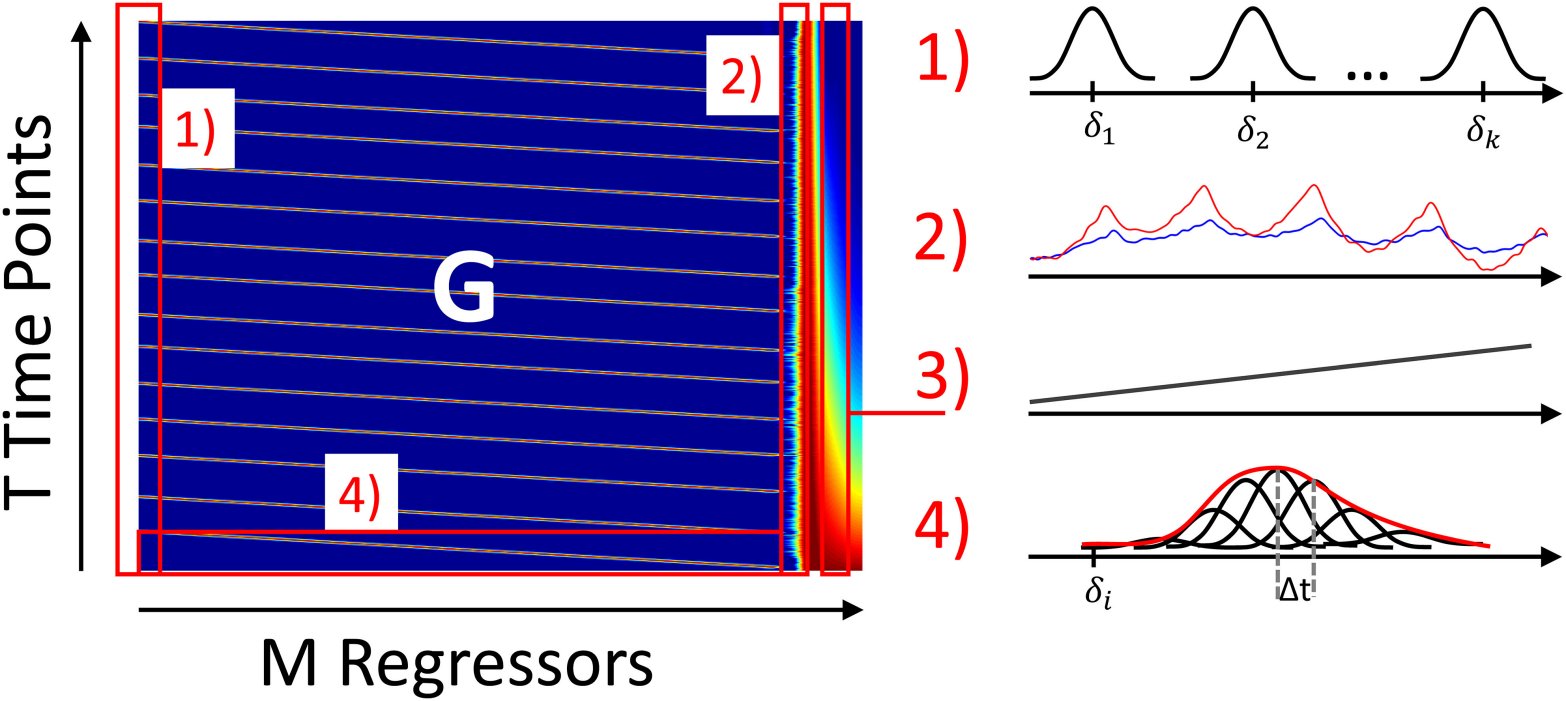
Visualization of the GLM design matrix G. (1): HRF regressor (Gaussian basis function) repeated at each stimulus. (2) Short-separation regressor. (3) Drift regressor. (4) Example GLM solution with all M weighted Gaussian bases forming the estimated HRF across trials hrf(t), see equation (3).

The current state-of-the-art fNIRS GLM approach uses polynomials to model *drift*_*n*_ and short-separation (SS) fNIRS measurements as regressors to model systemic physiology: $y_{n}^{physiology}\left(t\right)=\overset{J_{SS}}{\underset{j}{\sum }}\beta_{n,j}^{SS}y_{j}^{SS}\left(t\right)$. All regressors are combined to form the design Matrix G, which is visualized in Figure 5, and the GLM Equation (1) is solved for each regressor's contribution, the coefficients $\hat{\beta }$, in a linear least squares approximation:

(5) $$\begin{array}{l} \hat{\beta }={\left(G^{⊺}G\right)}^{-1}G^{⊺}\;Y. \end{array}$$

*The General Linear Model for fNIRS is an established supervised approach in neuroscience that combines a priori knowledge of experimental design and signal morphology. It provides the best linear unbiased estimate of the hemodynamic response to a series of stimuli in the presence of physiological nuisance signals*.

### 3.2. How to Incorporate the GLM Into Cross Validation Schemes

**Figure 6:**
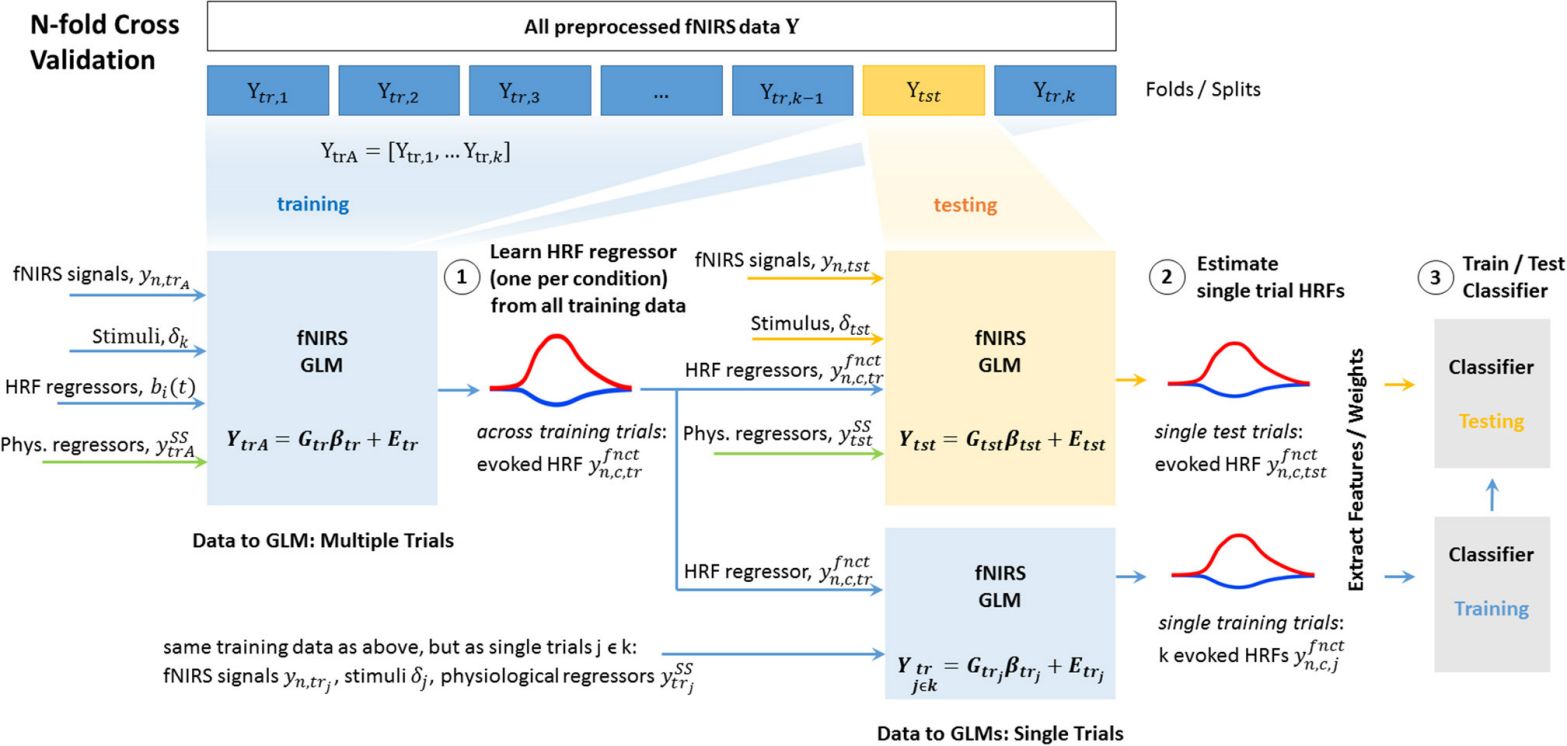
GLM for single trial analysis embedded in a cross validation pipeline. Steps 1, 2, and 3 are described in detail in section 3.2.

In its conventional application in neuroscience, the fNIRS GLM is being applied as a supervised approach to recover hemodynamic responses to different task conditions across all available trials. Consequently, solving Equation (5) to obtain the weights ($\hat{\beta }$) for the HRF basis functions and nuisance regressors yields a best estimate across the whole dataset, and the estimate improves by increasing the number of trials provided. Here, we describe our proposed way of incorporating the GLM into a cross validation scheme, when fNIRS signals are to be analyzed on a *single-trial basis*, specifically for the prediction and classification in BCI scenarios. The proposed approach ensures that preprocessing, training of the HRF shape and training and testing of the classifier maintain the integrity and separation of training and unseen testing data. The overall implementation is schematically depicted in Figure 6.

#### 3.2.1. Step 1: Learning the Individual and Channel-Specific HRF Shape From Training Trials

In each fold of an N-fold cross validation, the GLM is solved for each fNIRS channel *n* including all available training trials *trA* = [*tr*1, …*trk*] to find (1) weights $\hat{\beta }$ for the HRF basis functions *b*_*i*_(*t*) for each experimental condition *c* and (2) weights $\hat{\beta }$ for the regressors that model the physiological systemic nuisance signals $y_{trA}^{SS}$ and the polynomial drift. The GLM solution minimizes the sum of squared residuals between the linear sum of these model terms and the continuous fNIRS time series of that channel *y*_*n, trA*_. *y*_*n, trA*_ is the original time series signal excluding all sample points within the testing interval *tst*. The rows of the design matrix that correspond to the testing interval *tst* are also set to zero. This way the time structure of the data is kept intact while the GLM solution is obtained without including any information from the testing data. The result is the best linear unbiased estimate of the individual and channel-specific HRF, denoted as $y_{n,c,tr}^{fnct}$, estimated across training trials of an experimental condition *c*. It is the sum of the individually weighted basis functions *b*_*i*_(*t*) and their corresponding weights ${\hat{\beta }}_{HRF,i}$. This across-trial estimate is now used as an *individually learned HRF regressor* to assess single trial responses in step 2.

#### 3.2.2. Step 2: Obtaining Single Trial Estimates From Training and Testing Data

Using the learned HRF regressor $y_{n,c,tr}^{fnct}\;$for each condition, the GLM is now set up and solved individually for each trial in the training splits *tr, j* and testing split *tst*. In each trial, aside from the individual and channel-specific HRF regressor obtained as described in Step 1, the physiological nuisance regressors, (linear) drift regressors and the measured fNIRS signals in the trial's interval are sole inputs to the model. Each individual GLM solution yields an unbiased trial by trial estimate of how pronounced the previously learned hemodynamic response to a condition is in the presence of nuisance signals. The resulting estimate is expressed by the HRF regressor weight ${\hat{\beta }}_{HRF,c}$ for each condition. In scenarios with multiple conditions, the GLM is solved *c* times for each trial using the *c* available HRF regressors.

#### 3.2.3. Step 3: Feature Extraction, Training and Testing

Assuming stationarity, the estimated single trial HRF time signal can now be used for conventional feature extraction. If non-stationarities are to be taken into account for further identification and processing, the GLM's residual *E* can be added to the estimate. As an alternative to the extraction of conventional features from the estimated HRF time signal such as average or slope, the scalar regressor weight ${\hat{\beta }}_{HRF,c}$ itself can be used as a feature. Training and testing of the classifier is then performed conventionally using the single trial estimates for training and test trials, and steps 1–3 are repeated for each fold of the cross validation. Remarks: (1) Ideally, the individual HRF shape is learned in a training session previous to the actual BCI experiment. Step 1 can then be performed initially outside of the cross validation loop, which, however, requires more experimental data. (2) The described approach can easily be integrated into common existing offline-single trial analysis pipelines. To enable online single-trial analysis, e.g., online BCI, the GLM can be implemented in a state-space approach, for instance a Kalman filter, as was previously shown by Diamond et al. (2006) and Abdelnour and Huppert (2009). This approach will be briefly discussed in section Conclusion and Future Directions.

To integrate the GLM into a conventional cross validation pipeline for single-trial analysis requires:

1. *Learning the individual, channel and task specific HRF response across a set of training trials*.
2. *Obtaining the unbiased estimate of this HRF's weight in each single trial for both training and testing data. The estimated single trial HRF signal can then be processed conventionally for feature extraction, training and testing of a classifier*.

### 3.3. Evaluation

Here we compare HRF recovery performance, feature quality and classification performance for typical fNIRS-based BCI pre-processing vs. GLM with SS. In order to do so, we generate ground truth data and apply processing pipelines as detailed below for each method for the estimation of the evoked hemodynamic signal. We use functions from the established HOMER2 fNIRS toolbox (Huppert et al., 2009) for signal processing. In sections 4 and 5, we provide a quantitative comparison between the performance of typical fNIRS-based BCI preprocessing (will be denoted as “no-GLM” from now on) and the proposed integration of the GLM with SS.

#### 3.3.1. Synthetic HRF on Resting State Data

**Figure 7:**
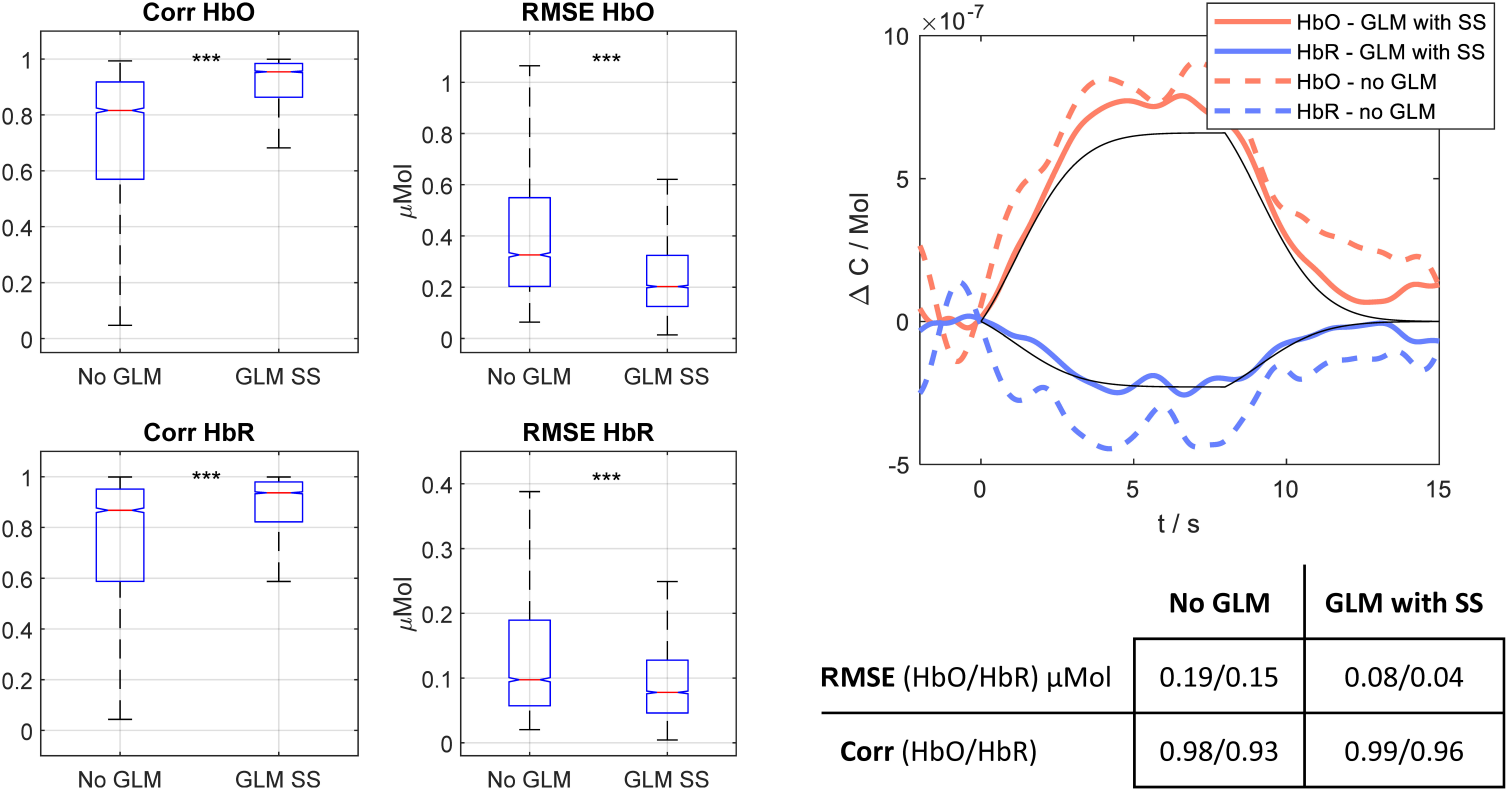
**(Left)** Correlation and RMSE boxplots for extracted single trial HRFs with both methodological approaches. Red line: median. Bottom/top box edges: 25th/75th percentile. Whiskers extend to most extreme data points that are not outliers. Significance for paired *t*-test ***p << 10^−3^. **(Right)** Exemplary HRF recovered with both approaches. HbO/HbR (coral/blue) estimated via GLM with SS (solid) and no-GLM (dashed). Table provides the RMSE and Corr values for GLM with SS and no-GLM for the depicted HRFs.

We generated ground truth data by augmenting fNIRS resting state data from 48 long-separation channels from 14 participants (see Appendix) with synthetic ground truth HRFs at randomized stimulus intervals. We generated a synthetic HRF following the *GAM* function in AFNI (Cox, 1996) which uses a gamma function convolved with a square wave. The resultant HRF has a time-to-peak of 6 s and a total duration of 15 s resulting in an increase in HbO of 0.66 μMol and a decrease in HbR of −0.23 μMol (Figure 7, right panel). The synthetic HRF is convolved with an onset vector with random inter-stimulus interval between 0 and 6 s and is then added onto a randomly selected half of the 48 available channels for the “STIM” condition. None of the channels were augmented with HRFs during the “REST” conditions. Overall, this yielded between 15 and 20 trials per condition.

#### 3.3.2. Comparative Signal Pre-processing and HRF Estimation With and Without GLM

##### 3.3.2.1. Common preprocessing for both methods

For both typical no-GLM (A) and GLM with SS (B), noisy fNIRS channels were identified and pruned using the HOMER2 function *hmrPruneChannels* (*dRange* = *10*^4^*-10*^7^ (corresponding to 80 and 140 dB for a TechEn System) and *SNRthresh* = 5). All fNIRS data is then converted into optical density and low pass filtered at 0.5 Hz with a zero-phase Butterworth filter of effective order 6. Optical density is then converted into concentration changes using the modified Beer-Lambert law with a partial pathlength factor of 6 (Delpy et al., 1988; Boas et al., 2004).

##### 3.3.2.2. Detrending

For training and testing splits of data, the signal was linearly detrended (A) for no-GLM by subtracting the linear least squares fit of each trial, and (B) by inserting a 1st order polynomial drift regressor term into the GLM with SS.

##### 3.3.2.3. HRF extraction

(A) The detrended concentration time course between the time period of 2 s prior to stimulus onset to 15 s after stimulus is used as the single trial HRF for no-GLM. (B) For the GLM with SS approach the HRF is modeled using a consecutive sequence of Gaussian functions with a standard deviation of 0.5 s and their means separated by 0.5 s (see section 3.1). The *hmrDeconvHRF_DriftSS* function in HOMER is then used to simultaneously estimate the HRF time signal together with the 1st order polynomial drift regressor term and one short-separation regressor term which corresponds to the signal at the fNIRS SS channel that has the highest correlation with the long-separation channel under investigation.

##### 3.3.2.4. Baseline correction

The single trial HRF is then baseline corrected using the mean of the signal from 2 s prior to the onset of the stimulus to the onset of the stimulus for both no-GLM and GLM with SS.

## 4. Performance in HRF Recovery and Feature Extraction With and Without GLM

With the ground truth data, we compare no-GLM and GLM with SS with respect to their recovery performance of fNIRS hemodynamic responses and feature quality. In the following, *recovered/estimated HRF* means the remaining fNIRS signal after preprocessing using these two approaches as detailed in section 3.3.2. Single trial recovery performance across an observed trial length *T* is evaluated in terms of:

1. the Pearson's correlation coefficient (Corr) between the estimated and ground truth HRF, obtained by using the “*corr*” function in MATLAB (MathWorks Inc., Natick, MA).
2. the root mean squared error (RMSE) between the estimated ($\hat{HRF}$) and ground truth HRF (*HRF*) time series is calculated as $RMSE=\;\sqrt{\frac{1}{T}\overset{T}{\underset{t=1}{\sum }}{\left(HRF\left(t\right)-\hat{HRF}\left(t\right)\right)}^{2}}$.

Figure 7 shows the boxplots and Table 1 shows the 1st/2nd order statistics for both metrics and chromophores for no-GLM and GLM with SS. For both metrics and across all trials, subjects and channels (a total of 3,960 recovered hemodynamic responses per chromophore and method), the results of the GLM with SS are significantly closer to ground truth than those yielded without GLM (*p* ≪ 10^−3^, paired *t*-test).

**Table 1:** 1st and 2nd order statistics of HRF recovery metrics Corr and RMSE.

	**(Mean ± Std.)**	**No-GLM**	**GLM with SS**
Corr	HbO	0.73 ± 0.25	0.90 ± 0.15
	HbR	0.75 ± 0.26	0.88 ± 0.17
RMSE (μMol)	HbO	0.47 ± 0.47	0.27 ± 0.29
	HbR	0.17 ± 0.21	0.10 ± 0.10

**Figure 8:**
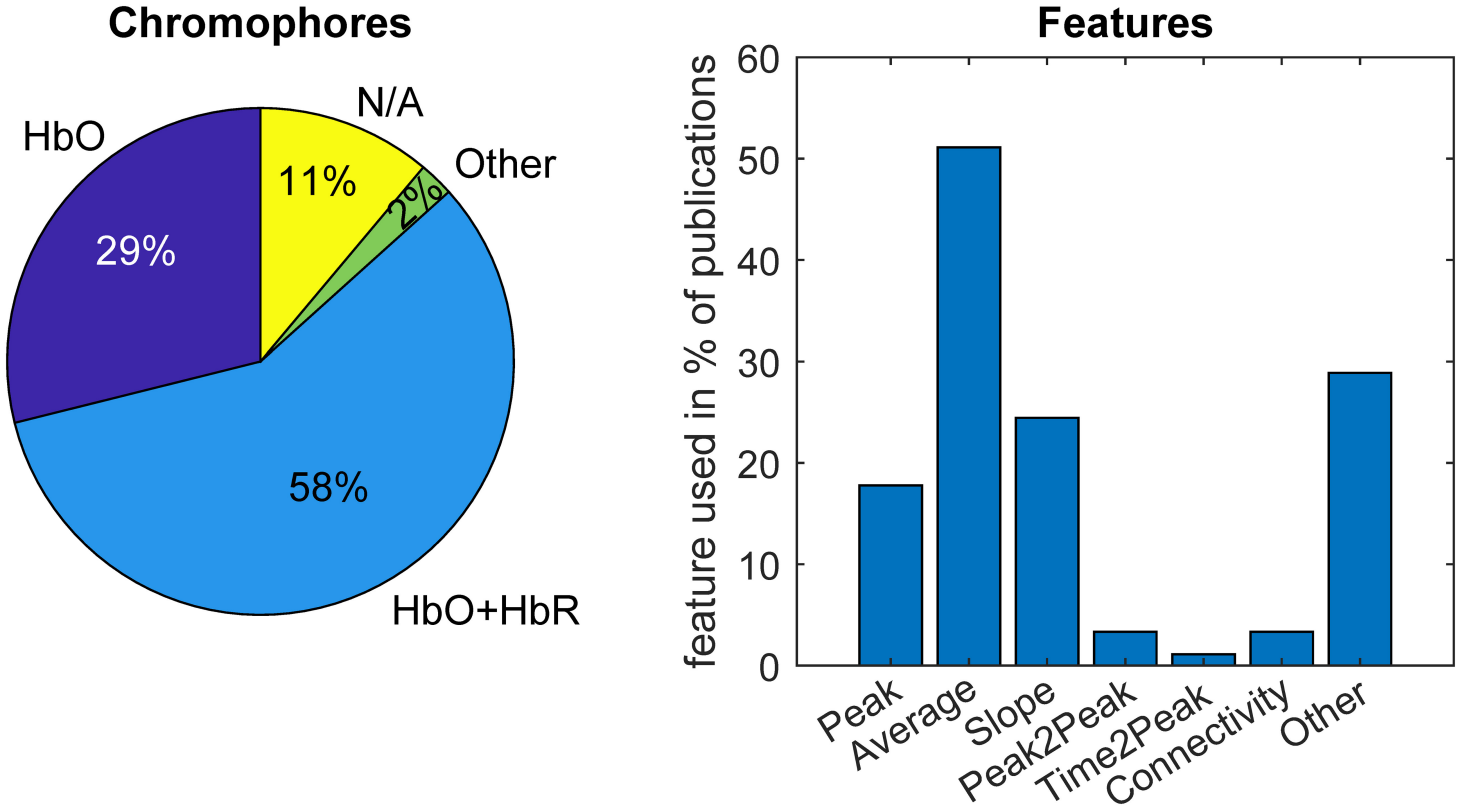
Chromophores and features typically used in fNIRS-based BCI literature. Percentages from top 100 most cited papers in Web of Science excluding review papers. Keyword search: fNIRS & BCI || fNIRS & Classification.

Both preprocessing approaches can also be contrasted in terms of the absolute errors between features extracted from the preprocessed single trial HRFs and from the synthetic HRF ground truth. For this comparison, we investigate the features most commonly used in BCI literature, also depicted in Figure 8. These are:

- Min/Max (Peak): Min (or max) is the minimum (or maximum) HbO (/HbR) amplitude within the estimated HRF time window.
- Peak to peak is the difference between the maximum and minimum HbO (/HbR) amplitude within the estimated HRF time window.
- Average is defined as the mean of HbO (/HbR) within the estimated HRF time window.
- Slope is the slope of a linear least squares fit to a pre-defined time window of the estimated HRF (HbO/HbR), here (0–4 s).

Other features include connectivity metrics, time to peak, and higher order statistics, such as skewness and kurtosis of the signal within an epoch.

**Figure 9:**
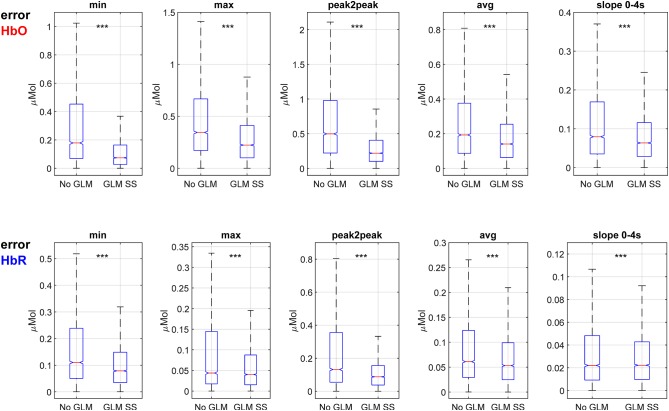
Boxplots of errors between ground truth features and features extracted from fNIRS signals after conventional preprocessing (no-GLM) and GLM with SS. ***p << 10^−3^.

Figure 9 shows boxplots of the errors between ground truth features and features extracted from the 3,960 estimated single trial HRFs using (A) the conventional pipeline (no-GLM) and (B) the GLM with SS. Across all trials, subjects and channels and for all feature types and chromophores, the error for GLM with SS is significantly smaller than for the no-GLM approach (*p* ≪ 10^−3^, paired *t*-test).

*The GLM with SS recovers the evoked hemodynamic brain signal by simultaneously estimating the contributions of HRF, physiological nuisance, and drift. This approach leads to a better estimate of the HRF time signal than conventional single trial BCI preprocessing pipelines, and consequently also improves the quality of features*.

## 5. Single Trial HRF Detection and Classification

**Figure 10:**
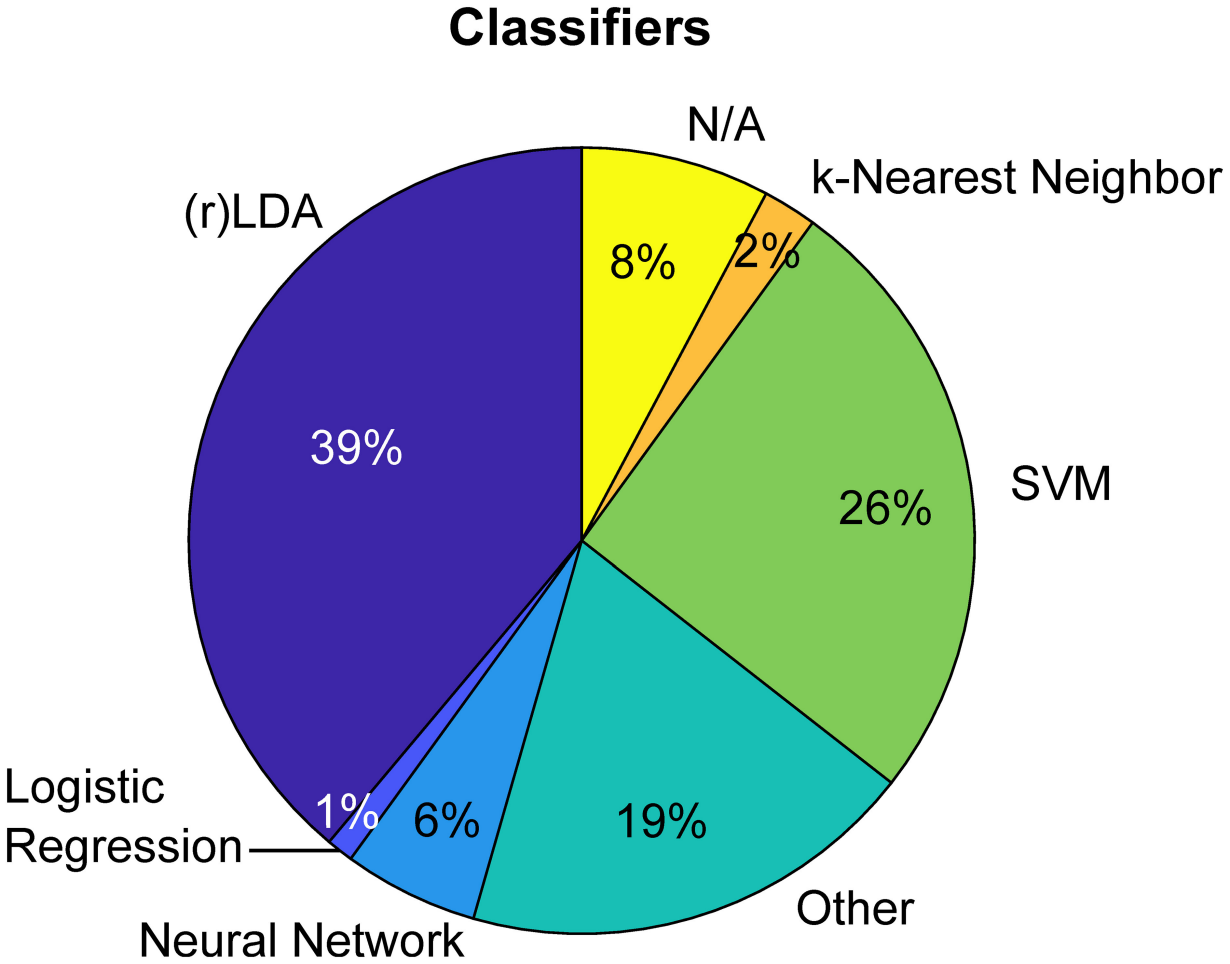
Commonly applied classifiers in fNIRS BCI research (top 100 most cited papers in Web of Science excluding reviews. Keyword search: fNIRS & BCI || fNIRS & Classification).

Single trial analysis pipelines typically aim to detect evoked brain responses to single events—and to discriminate between events of different conditions. In BCI, where the recovered brain signals are used to predict the condition (class) of an event, a standard approach is to determine the signal's statistical properties with the help of machine learning to train classifiers for the prediction. Most common among recent fNIRS BCI studies are classifiers based on regularized Linear Discriminant Analysis (rLDA, 39%) and Support Vector Machines (SVM, 26%) (see Figure 10). While the performance of a classifier is strongly dependent on model and feature selection, it ultimately depends on the presence of discriminative characteristics that are to be extracted from the signal. Clearly, a preprocessing pipeline that increases the evoked signal's contrast to noise ratio can play a significant role in the achievable predictive performance. In this section we briefly compare the impact of the two preprocessing approaches (typical pipeline: no-GLM, and proposed approach: GLM with SS) on the discriminability of the most commonly used features in fNIRS-based BCI studies and the resulting classification accuracy.

**Figure 11:**
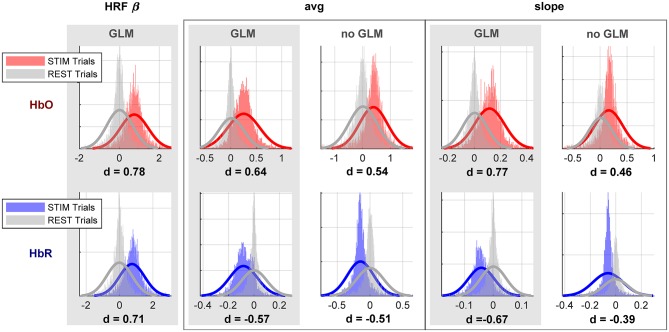
Histograms and Gaussian fits of selected feature types for both conditions (stimulus and rest) extracted from fNIRS signals preprocessed with conventional pipeline (no-GLM) and GLM with SS. Features are pooled across all subjects, channels and trials of the same condition. (Top): HbO, (bottom): HbR. d is the effect size expressed through Cohen's d.

Figure 11 shows the pooled distributions of the two most commonly applied feature types, average and slope, as well as our newly proposed feature type, the HRF regressor weight “β,” across all subjects, channels and trials. We express the effect size of the distribution's mean differences by *Cohen*′*s d*: = (μ1 − μ2)/*s* with *s* being the pooled stadard deviation. The effect size of the mean distance between the distributions for stimulus vs. rest conditions is significantly larger for features that were extracted after GLM with SS preprocessing, indicating a better separability compared to preprocessing without GLM.

A typical measure of separability of a feature is the point biserial correlation coefficient (r^2^ value), which is defined as

(6) $$\begin{array}{l} r^{2}\left(x,y\right)=\;\frac{N_{1}\cdot N_{2}}{{\left(N_{1}+N_{2}\right)}^{2}}\frac{{\left(\mu_{1}-\mu_{2}\right)}^{2}}{var\langle x_{i}\rangle } \end{array}$$

with μ_*i*_ = *mean* 〈*x*_*i*_〉_*yi* = 1_ being the class means and *N*_*k*_ = |{*i* | *y*_*i*_ = *k*}| being the number of samples of class *k, x*_*i*_ the sample and *y*_*i*_ the class label of sample *i*.

For both preprocessing approaches, Table 2 summarizes the across-subject average r^2^ for each feature type and chromophore, each calculated across all augmented channels. GLM with SS preprocessing yields significantly higher biserial correlation coefficients for all compared features and chromophores (*p* < 2 × 10^−3^).

**Table 2:** Across subject average point biserial correlation coefficient per feature and preprocessing approach.

**r**^**2**^ **Mean ± Std**.		**HRF β**	**Min**.	**Max**.	**Peak to peak**	**Avg**.	**Slope**
No GLM	HbO		0.076 ± 0.058	0.068 ± 0.048	0.057 ± 0.054	0.107 ± 0.067	0.102 ± 0.068
	HbR		0.077 ± 0.072	0.081 ± 0.081	0.062 ± 0.070	0.110 ± 0.096	0.102 ± 0.081
GLM with SS	HbO	0.165 ± 0.097	0.108 ± 0.068	0.106 ± 0.075	0.132 ± 0.089	0.163 ± 0.101	0.166 ± 0.101
	HbR	0.166 ± 0.124	0.101 ± 0.071	0.112 ± 0.086	0.132 ± 0.112	0.166 ± 0.124	0.166 ± 0.124
Paired *t*-test (*p*-value)		N/A	<2 × 10^−3^	<10^−3^	<10^−3^	≪10^−3^	≪10^−3^

**Figure 12:**
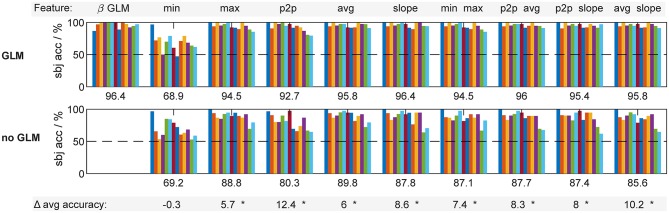
Classification results for each feature type and preprocessing method. Single bars (colors) are individual subjects. Labels under each group are average accuracies across subjects. Bottom numbers highlighted by gray bar are average differences between methods. Asterisks (*) indicate significant difference (paired *t*-test, *p* < 5%).

The enhanced separability of features from hemodynamic responses that are recovered with the GLM with SS naturally also improves classification performance. To exemplify this, we performed a typical classification approach, discriminating between resting vs. stimulation trials in a 10-fold cross validation using regularized Linear Discriminant Analysis with automatic shrinkage of the covariance matrices (Ledoit and Wolf, 2004; Blankertz et al., 2011). Within each fold, automatic feature selection was performed by choosing the 25 features with the highest r^2^ value among the training data. The pipeline was repeated for each available single feature and common two-feature combinations, and each preprocessing method. Figure 12 shows the resulting classification accuracies for each subject, feature and method, as well as the across-subject average performance.

Across all feature types and subjects, preprocessing the fNIRS data with the GLM with SS improved classification accuracy on average by 7.4% compared to the performance achieved with conventional preprocessing without GLM but otherwise identical classification pipeline and processing parameters. The new feature type, the HRF weight β, yielded the highest performance, identical to that achieved with the slope feature. It is notable that the HRF weight β inherently combines the characteristics of the other features, as it represents the strength of the individually trained whole HRF time course.

*The improved Contrast to Noise Ratio of HRFs recovered with the GLM can significantly reduce noise in the feature space of both chromophores, leading to improved separability of features and better classification performance*.

## 6. Conclusion and Future Directions

With the concurrent advances in wearable imaging systems and active and passive Brain Computer Interfaces in the last two decades, the exploration of signal processing for a robust estimation of brain activity has become increasingly vital. A successful BCI application requires robust feature extraction from brain signals that accurately reflect the intent/state of the BCI user (He et al., 2012) and not physiological artifacts. In fNIRS-based BCIs, obtaining such features heavily depends on the robust estimation of the underlying cerebral hemodynamic changes associated with the brain activity. fNIRS measurements, though, can be heavily contaminated by signals of physiological and/or non-physiological origin, especially when acquired in environments with more ecological validity, recently enabled by new wearable systems. Despite the availability of dedicated preprocessing methods that can successfully filter such confounding signals, the fNIRS BCI field is still in an early stage in exploring and adapting these approaches from conventional neuroscience. In this paper, we aimed to highlight the importance of proper preprocessing of fNIRS signal, and showed how using the state-of-the-art approach, i.e., the General Linear Model with short-separation regression, can improve the discriminative power of features and the resultant classification accuracy.

### 6.1. GLM in Single Trial Analysis—Considerations and Caveats

The General Linear Model allows simultaneous extraction of the evoked HRF response and filtering out confounding signals with the help of nuisance regressors. Independent measures of such nuisance factors, such as short-separation fNIRS measurements, have been shown to be quite effective in modeling the systemic interference and allow a more robust estimation of brain activity (Gagnon et al., 2011; Yücel et al., 2015). We have shown that the single trial hemodynamic signals recovered with the GLM are significantly closer to ground truth compared to the ones obtained with conventional fNIRS-based BCI preprocessing pipelines in terms of both the correlation and the root mean squared error. We should note that our simulated data did not involve any task-evoked systemic changes in the short and long-separation measurements. In an actual scenario, the task at hand can produce hemodynamic changes in the superficial layers that are time-locked to the cerebral hemodynamic changes but are not brain signal. In such cases, discriminative systemic physiology can increase the classification accuracy, as the classifiers use all the information at hand to obtain highest discriminative power. However, they can dramatically reduce the performance in real world settings where systemic physiology is more susceptible to spontaneous changes outside of the constrained paradigm. Examples are increases in motion related artifacts in the signal while running, or changes in scalp hemodynamics during different emotional states. This issue further emphasizes the importance of proper cleaning of physiological and non-physiological confounding factors in the signal.

The improved HRF recovery performance of the proposed approach also impacts the discriminative power of the resultant features and classification accuracy. All commonly used features in the fNIRS BCI field, namely peak, average, slope, and peak-to-peak, extracted from HRFs obtained via GLM with SS were significantly closer to the ground truth and more discriminative between classes, as expressed by increased Cohen's d and biserial correlation coefficients. Expectably, the increased discriminability also resulted in an increased classification accuracy. Alongside with commonly used features in the BCI field, we introduced a new feature type by obtaining an individual and channel-specific hemodynamic response function from the training data which essentially incorporates the information of all commonly used features. The estimation of the contribution of this individual and channel-specific hemodynamic response function in each single trial, as performed by the GLM, yields one single comprehensive feature—the HRF weight β. Such an approach not only provides the highest classification accuracy but also reduces the risk of a biased or suboptimal choice of feature types among the many available.

One caveat of using individual and channel-specific HRF regressors is that the inclusion of channels that show no activation differences across conditions (e.g., STIM/REST) can result in a feature distribution that impairs classification accuracy. While without a GLM approach the features obtained from non-active channels during STIM and REST condition have a random distribution, using an individually learned HRF regressor in the GLM approach forces the single trial HRF estimates in these channels to have a fixed shape, varying only by amplitude between trials. An HRF regressor learned from random signals in the STIM condition applied to random signals in the REST condition can consequently yield more similar features than would have been obtained on a random signal without GLM. These “false positives” can reduce discriminative power and classification accuracy. A simple but important remedy is to perform channel and/or feature selection: Common approaches are (1) applying a statistical test on the training data and pick the channels that show significant difference between conditions, thereby excluding inactive channels from the analysis. (2) Performing feature selection based on their separability, e.g., by means of a point biserial correlation coefficient, as done in this paper.

Accuracy and speed are two important performance measures in BCI applications, typically expressed together as the “information transfer rate” (ITR), in bits per minute or bits per trial (Wolpaw et al., 1998). The ITR naturally depends on the number of trials necessary (speed) for a robust estimation of the brain signal (accuracy). While using more than one trial improves the HRF estimation performance by better conditioning the design matrix in a GLM approach, here we showed that good performance and high classification accuracy can be achieved at the single trial level as well. By employing the GLM for single-trial analysis within cross validation as we propose in this manuscript, accuracy is increased by (1) obtaining the features from an individual and channel-specific HRF that was obtained from the training data which includes multiple trials and (2) properly removing the physiological and non-physiological nuisance from the test signal. Especially in passive BCI applications where speed can be less relevant and multiple trials can be analyzed simultaneously, it can be expected that the GLM approach improves accuracy even more beyond that achieved with conventional preprocessing.

### 6.2. Further Improved Nuisance Regression—Using the GLM With tCCA Regressors

We have recently shown that the presented state-of-the-art GLM using short-separation measurements as nuisance regressors can be further improved by incorporating temporally embedded Canonical Correlation Analysis (tCCA) (von Lühmann et al., 2020). tCCA enables the combination of any available auxiliary signals, including short-separation signals, accelerometer, respiration, blood pressure, and others, into more optimal nuisance regressors. By temporally embedding the auxiliary signals, a remedy to non-instantaneous and non-constant coupling between auxiliary and fNIRS signals is provided (von Lühmann et al., 2019). This makes the GLM solution less susceptible to errors from varying time delays between physiological nuisance signals in the measurement channels. The new approach models the physiological component in Equation (2) as $y_{n}^{physiology}\left(t\right)=\overset{J_{CCA}}{\underset{j}{\sum }}\beta_{n,j}^{CCA}{\hat{s}}_{j}^{CCA}\left(t\right)$, where ${\hat{s}}_{j}^{CCA}$ are the *J*_*CCA*_ regressors found by optimizing the tCCA objective function. The so found nuisance regressors improve GLM performance significantly in terms of the recovered HRF shape and Contrast to Noise Ratio as well as sensitivity and specificity, especially when the number of available trials are low (von Lühmann et al., 2020). tCCA regressors can be determined as a common set of multiple regressors for all fNIRS channels, or one individual tCCA regressor per fNIRS channel. The GLM with tCCA approach can be easily integrated into the proposed (cross validated) preprocessing pipeline for single-trial analysis in this manuscript. Instead of using *J*_*SS*_ short-separation channels $y_{j}^{SS}$, one simply employs the new *J*_*SS*_ tCCA regressors ${\hat{s}}_{j}^{CCA}$ for physiological regression in the GLM. There are two additional requirements: (1) The experimental setup has to include the acquisition of the additional auxiliary signals, with SS fNIRS channels and accelerometer being the most valuable, and (2) a few minutes of individual resting state data without evoked hemodynamic responses are required to train the tCCA projection filters. While the tCCA approach adds some complexity, it provides solutions for the challenges arising in real-world fNIRS application scenarios, such as BCI, neuroimaging and Neuroergonomics out of the lab, where physiological interference can otherwise be a major hindrance to robust single trial analysis (von Lühmann et al., 2019).

### 6.3. Real Time Implementation of the Proposed GLM Approach

In conventional fNIRS and fMRI neuroscience, the GLM is usually applied to supervised offline analysis of multiple trials at once. In this manuscript, we showed how to adapt it to single trial analysis within a cross validation scheme. Several ways exist to achieve real time capability of this approach. One way is to use a Kalman filter based state-space modeling approach. The Kalman filter method is a dynamic tracking scheme that estimates the state *x*_*n*_ of a process using a recursively updated regularized linear inversion routine. It has been successfully adapted for the fNIRS GLM by others and us in the past to adaptively estimate the GLM coefficients β for each time step (Diamond et al., 2006; Abdelnour and Huppert, 2009; Hu et al., 2010; Gagnon et al., 2011). Building on these previous adaptations allows the straight-forward integration of both the SS regression and tCCA-based noise regressors to achieve a robust real-time estimation of the GLM coefficients and evoked hemodynamic responses.

BCI and more integrative human-machine interfaces (HMI) that use both brain and body signals, have unprecedented potential to improve healthcare, work environments, efficiency, and security and can advance our understanding of brain function and cognition in general and especially under everyday life conditions. For the transition from well-controlled laboratory environments into real life applications, the robust separation of task-evoked brain activity from a wide range of confounding physiological and non-physiological nuisance factors is required. In fNIRS-based BCIs, a General Linear Model approach with short-separation regression and an individual and channel specific-HRF model obtained from a training data set can increase performance. By simultaneously estimating systemic physiology and evoked brain responses, it improves features separability and classification accuracy even at a single trial level.

## Data Availability Statement

All the relevant code is currently available on https://github.com/avolu/GLM-BCI with open public access. The data used in this work is de-identified according the guidelines of the Institutional Review Board of Boston University and will be provided upon request.

## Ethics Statement

The study has been reviewed and approved by the Institutional Review Board of Boston University. The patients/participants provided their written informed consent to participate in this study.

## Author Contributions

AL, MY, and AO-M developed the concept of this work, performed the analysis, designed the figures, and drafted the manuscript. DB provided critical feedback on the concept and results. AO-M created the database of 100 most cited fNIRS-BCI papers. All authors read and approved the final manuscript.

### Conflict of Interest

The authors declare that the research was conducted in the absence of any commercial or financial relationships that could be construed as a potential conflict of interest.

## Supplementary Material

Supplementary Table 1 Preprocessing steps applied by the top 100 most-cited papers in Web of Science within the fNIRS-BCI field (Search words: fNIRS & BCI or fNIRS & Classification, search year: 2019).

## A. APPENDIX

### A.1. fNIRS Resting State Data

Ten minutes of fNIRS resting state data were collected from 13 healthy subjects (age: 30 ± 17 years; 7 male/5 female/1 not reported) who gave their signed written informed consent form prior to the experiment. The study was approved by and carried out in accordance with the regulations of the Institutional Review Board of Boston University. Subjects had no neurological or psychological disorders.A CW7 fNIRS system (TechEn Inc. MA, USA) with 32 frequency-encoded lasers (half at 690 and half at 830 nm) and 32 avalanche photo-diode detectors was used for data acquisition. The sample rate was 50 Hz. Subjects were seated in a comfortable chair and an fNIRS probe was placed on their head. The head probe was consisted of an elastic cap (EasyCap, Herrsching, Germany) with 16 sources, 24 long-separation detectors (~30 mm apart from the source) and 8 short separation detectors (~8 mm apart from the source) providing, in total, 48 long-separation and 8 short-separation channels.
